# Evaluation of the uncertainty in an EBT3 film dosimetry system utilizing net optical density

**DOI:** 10.1120/jacmp.v17i5.6262

**Published:** 2016-09-08

**Authors:** Elsa Y. León Marroquin, José A. Herrera González, Miguel A. Camacho López, José E. Villarreal Barajas, Olivia A. García‐Garduño

**Affiliations:** ^1^ Laboratorio de Fotomedicina, Biofotónica y Espectroscopia Láser de Pulsos Ultracortos, Facultad de Medicina, Universidad Autónoma del Estado de México Jesús Carranza y Paseo Tollocan s/n. Toluca México; ^2^ Laboratorio de Física Médica, Instituto Nacional de Neurología y Neurocirugía Mexico City México; ^3^ Departamento de Oncología & Departamento de Física y Astronomía Universidad de Calgary Calgary AB Canada

**Keywords:** radiochromic film, flatbed scanner, dosimetry, uncertain analysis

## Abstract

Radiochromic film has become an important tool to verify dose distributions for intensity‐modulated radiotherapy (IMRT) and quality assurance (QA) procedures. A new radiochromic film model, EBT3, has recently become available, whose composition and thickness of the sensitive layer are the same as those of previous EBT2 films. However, a matte polyester layer was added to EBT3 to prevent the formation of Newton's rings. Furthermore, the symmetrical design of EBT3 allows the user to eliminate side‐orientation dependence. This film and the flatbed scanner, Epson Perfection V750, form a dosimetry system whose intrinsic characteristics were studied in this work. In addition, uncertainties associated with these intrinsic characteristics and the total uncertainty of the dosimetry system were determined. The analysis of the response of the radiochromic film (net optical density) and the fitting of the experimental data to a potential function yielded an uncertainty of 2.6%, 4.3%, and 4.1% for the red, green, and blue channels, respectively. In this work, the dosimetry system presents an uncertainty in resolving the dose of 1.8% for doses greater than 0.8 Gy and less than 6 Gy for red channel. The films irradiated between 0 and 120 Gy show differences in the response when scanned in portrait or landscape mode; less uncertainty was found when using the portrait mode. The response of the film depended on the position on the bed of the scanner, contributing an uncertainty of 2% for the red, 3% for the green, and 4.5% for the blue when placing the film around the center of the bed of scanner. Furthermore, the uniformity and reproducibility radiochromic film and reproducibility of the response of the scanner contribute less than 1% to the overall uncertainty in dose. Finally, the total dose uncertainty was 3.2%, 4.9%, and 5.2% for red, green, and blue channels, respectively. The above uncertainty values were obtained by minimizing the contribution to the total dose uncertainty of the film orientation and film homogeneity.

PACS number(s): 87.53.Bn

## I. INTRODUCTION

Initially, the EBT and EBT2 Gafchromic film models were designed for intensity‐modulated radiotherapy (IMRT) and quality assurance (QA) procedures.^1^, ^2^, ^3^, ^4^, ^5^, ^6^ Currently, radiochromic film dosimetry protocols developed for IMRT QA have suggested the use of flatbed scanners to read the radiochromic film.^3^, ^5^, ^7^, ^8^, ^9^ The dosimetry system consists of the radiochromic film and the flatbed scanner. This dosimetry system is used for two‐dimensional measurements of dose distributions in radiotherapy applications such as IMRT and stereotactic radiosurgery. The system allows one to perform dose distributions measurements with high spatial resolution (about 1200 lines/mm). However, certain factors contribute to the total uncertainty in determining dose. Therefore, considering and minimizing these factors is very important to ensure that the uncertainty for each treatment technique is acceptable. The factors to consider include the dependence of the response on the relative orientation of the film scanner, the lack of uniformity in the useful scan area, the scan parameters, the scanner stability, the scanner uncertainty, and curve calibration.^10^, ^11^, ^12^, ^13^, ^14^ To this end, the dose measurements with the EBT film using the red channel when the film is read with a flatbed scanner have been rigorously investigated because absorption is highest at 636 nm.^15^, ^16^ The dynamic range of the EBT film in the red channel reaches a maximum of approximately 8 Gy,^(17)^ but higher dose ranges result in the saturation of the red channel response curve.

Over time, radiochromic films have been employed in other dosimetry applications, including brachytherapy, skin dose measurement, lung and breast phantom measurements, total body irradiation (TBI), total skin electron therapy (TSET), electron therapy, stereotactic radiotherapy, the dosimetry characterization of proton therapy beams, and dose verification during cell irradiation in radiobiological experiments.^18^, ^19^, ^20^, ^21^, ^22^, ^23^, ^24^, ^25^, ^26^, ^27^, ^28^, ^29^ These applications necessitate the study and characterization of the EBT3 radiochromic film for dose ranges above 8 Gy.

The aim of this paper was to analyze and evaluate the dosimetry system formed by the EBT3 radiochromic film and Epson Perfection V750 for doses ranging from 0 to 120 Gy using three color channels. Furthermore, an uncertainty analysis of the dose was performed to study certain intrinsic characteristics of film dosimetry.

## II. MATERIALS AND METHODS

### A. Radiochromic film

The radiochromic film used in this study was a Gafchromic EBT3 (Gafchromic, International Specialty Products, Wayne, NJ) film with serial number A01171301 and sheet dimensions of 20.3 cm×25.4 cm. The sheets were cut into pieces of 3 cm×3 cm for all experiments, and the films were handled and used according to the general recommendations outlined by the manufacturer's specifications^(17)^ and AAPM TG‐55.^(30)^ Radiochromic EBT3 film consists of a single active layer, nominally 27 mUm thick, between two transparent polyester substrates with a thickness of 120 μm each.^(17)^ The active layer contains the active component, marker dye, stabilizers, and other additives, giving the film its low‐energy dependence. The active layer of EBT3 radiochromic films consists of H (56.8%), C (27.6%), O (13.3%), Al (1.6%), and Li (0.6%). Therefore, its effective atomic number is 7.26, according to the manufacturer. The EBT3 film model presents some improvements, such as greater uniformity, less than Active layer incorporates a yellow marker dye to decreases UV/light sensitivity and enables all the benefits of multichannel dosimetry, when it's used in conjunction with an RGB film scanner. The symmetric structure eliminates the need for keeping track of which side of the film is facing the light source of the scanner. The polyester substrate has a special surface treatment containing microscopic silica particles that maintain a gap between the film surface and the glass window in a flatbed scanner. Since the gap is nearly ten times the wavelength of visible light, formation of Newton's Rings interference patterns in images acquired using flatbed scanners is prevented.^(17)^

### B. Irradiation film procedure

Each film piece was placed at a 5 cm depth in a solid water phantom (CIRS Inc., Norfolk, VA), which consisted of 30 cm×30 cm slabs of different thicknesses. The total thickness of the phantom was 30 cm. The films were perpendicularly irradiated with a 6 MV photon beam using a Novalis linac linear accelerator (Brainlab AG, Feldkirchen, Germany). The linac was calibrated such that a 1 cGy per monitor unit was delivered at a 5 cm depth with a 10 cm×10 cm field size and a source‐to‐surface distance (SSD) of 95 cm. The films were exposed to various absorbed doses ranging from 0 to 120 Gy to cover the full dynamic range of the film considering the following intervals: from 0 to 0.5 Gy in steps of 0.25 Gy, from 0.5 to 3 Gy in steps of 0.5 Gy, from 3 to 10 Gy in steps of 1 Gy, from 10 to 50 Gy in steps of 5 Gy, and from 50 to 120 Gy in steps of 10 Gy. In order to reduce the statistical uncertainty, each calibration point consisted of five irradiated film pieces.^(31)^ A total of 150 film pieces were used to build the sensitometric curve.

### C. Scanning protocol and analysis

In this study, an Epson Perfection V750 desktop flatbed scanner (US Epson, Long Beach, CA) and its associated software, Epson Scan, were used to read all films before and after irradiation. To minimize the effect of the nonuniform response of the readout due to the light scattering of the scanner lamp caused by particles in the film active layer,^(32)^ a cardboard template was fitted to the scanner to position films at a reproducible central location of the scan surface that can be considered uniform.^(17)^ The films were scanned after a 15 min warm‐up time to stabilize the flatbed scanner, according to Ferreira et al.^(33)^ Images were acquired in transmission mode, landscape orientation, and RGB‐positive mode at a depth of 16 bits per color channel with a spatial resolution of 72 dpi, which corresponded to a pixel size of 0.35 mm×0.35 mm. The images were saved in .tiff format.

The raw images of films were imported from the scanning system into the #acm20001xImageJ (v.1.2) analysis software for further image processing to obtain the values of the transmitted light intensity (I) and standard deviation associated with this value (SD(I)). The images were processed in three colors channels (red, green, and blue).

### D. Film response

The physical principle of radiochromic film is a color change in response to radiation exposure. Therefore, the response of the EBT3 radiochromic film is characterized by the net optical density (netOD). The net optical density (netOD) is related to the intensity (I) by the Lambert‐Beer law,^(34)^ as indicated by the following equation:

(1) $$netOD=–log_{10}\frac{I}{I_{0}}$$

where *I_0_* and *I* are the reading for the unexposed and exposed film piece, respectively. To homogenize the film response, a correction procedure was performed, accordingly to Garcia‐Garduno et al.^(35)^

Conversely, using the error propagation expression and ignoring cross‐correlations,^(36)^ obtain the associated standard deviation net optical density (SD(netOD)) using the following equation:

(2) $$SD(netOD)=\frac{1}{ln\;10}\sqrt{{\left(\frac{SD(I_{0})}{I_{0}}\right)}^{2}+{\left(\frac{SD(I)}{I}\right)}^{2}}$$

where $SD(I_{0})$ and *SD(I)* are the associated standard deviations $I_{0}$ and I, respectively.

### E. Experiments to characterize the dosimetry system

#### E.1 Dynamic range

Radiochromic EBT3 film is designed to be used in a wide range of doses when analyzed with a flatbed scanner using three color channels: red, green, and blue.^(17)^ In this study, the dynamic range of the radiochromic film was determined by analyzing the sensitivity and uncertainty of the film response.^(37)^ The radiochromic film was irradiated from 0 to 120 Gy to ensure that covered the entire dynamic range. With the information obtained from the image analysis, the response curves of the film were constructed by plotting the net optical density as a function of dose. These curves were fitted to a power function of the following form:

(3) $$netOD=aD+bD^{n}$$

where *a, b*, and *n* are fitting parameters and *D* is the measured dose in Gy. Subsequently, the response sensitivity of the film EBT3 for each color channel, which is defined as the derivative of the slope of the calibration curve at each point,^(38)^ was calculated. In mathematical form, this sensitivity is given by the following:

(4) $$S=\frac{dnetOD}{dD}=a+nbD^{n-1}$$

The sensitivity curves of radiochromic EBT3 film were used to determine the dynamic range for each color channel based on two points of intersection. The first point is the intersection of the sensitivity curves of the red and green channels ($S_{\text{RG}}$), and the second point corresponds to the intersection of the sensitivity curve of the green channel with blue channel curve ($S_{\text{GB}}$).

#### E.2 Response curves and fitting procedure

The radiochromic film was used for dose measurement, but the dose is more conveniently plotted as a function of the measured net optical density, allowing the data to be fitted to a curve. This analytical curve was used to convert the measured net optical density to dose values.

To obtain the dose response relationship, we used the pieces of EBT3 film irradiated at different dose levels between 0 and 120 Gy. The analytical expression was obtained by fitting the data using the least squares method; a potential equation such as Eq. (3) with the dose as the dependent variable is given by the following:

(5) $$D=anetOD+bnetOD^{n}$$

Dose‐response curves were obtained for the three color channels by considering the dynamic ranges determined in this study for each color channel (see Materials and Methods section E.1).

The uncertainty in determining the dose was calculated using an error propagation analysis, as proposed by Devic et al.^(39)^ The total scan uncertainty (${\text{SD}}_{\text{tot}}$) was calculated using the following expression:

(6) $$SD_{tot}=SD_{exp}^{2}+SD_{fit}^{2}$$

where *SD_exp_* is the experimental uncertainty and resulted the uncertainties associated with the film irradiation and scanning procedures. Furthermore, ${\text{SD}}_{\text{fit}}$ represents the fitting uncertainty. The mathematical expressions of ${\text{SD}}_{\text{exp}}$ and ${\text{SD}}_{\text{fit}}$ are

(7) $$SD_{exp}(\%)=\frac{(a+n\cdot b\cdot netOD^{n–1})\cdot SD(netOD}{D_{fit}}\cdot 100$$

(8) $$SD_{tot}(\%)=\frac{netOD^{2}\cdot SD_{a}^{2}+netOD^{2n}\cdot SD_{b}^{2})}{D_{fit}}\cdot 100$$

where ${\text{SD}}_{a}$ and ${\text{SD}}_{b}$ are the fitting parameter uncertainties, and *SD(netOD)* is the uncertainty associated with the measured optical density calculated within Eq. (2).

#### E.3 Dose resolution of the system

The definition of dose resolution used in this work was taken from Baldock et al.^(40)^ and it is defined as the minimal separation of two absorbed doses at which their most probably value is different with a given level of confidence. The dose resolution ($D_{R}$) of the measurement system was calculated from the response curve (Materials and Methods section E.2) by multiplying the standard deviation of netOD (SD(netOD)) associated to each dose level times the value of the first derivative of the fit function at the respective dose point.

(9) $$D_{R}=SD(netOD)\cdot \frac{dD}{dnetOD}$$

The dose resolution of the system was evaluated in the dose range from 0 to 120 Gy.^(5)^

### F. Experiments to characterize the Gafchromic EBT3 film

#### F.1 Film reproducibility and uniformity

To study the radiochromic EBT3 film reproducibility, the standard deviation (SD) of the response (net optical density) was calculated using Eq. (2) for 165 film pieces of 3 cm×3 cm irradiated between 0 and 120 Gy. These pieces were cut from different regions of five film sheets.

The uniformity of the radiochromic EBT3 film was investigated by comparing the dose measured in ROIs at different locations on a single sheet of the film. Ten pieces of the same film had been irradiated at 0, 1, 6, 15, 35, and 70 Gy. The uncertainty due to film uniformity was defined as the standard deviation from the mean in the ROI of each film piece.

#### F.2 Relative orientation of the film

To determine the influence of the relative orientation of the film during scanning, the 165 film pieces irradiated from 0 to 120 Gy were scanned both in landscape and portrait orientation. The expression portrait orientation is used when the scanning direction is perpendicular to the shorter leaf film side, while the expression landscape orientation is used if both directions are parallel. The reason is that, in these films, the polymers of the active layer are as small needles, thereby changing the response of the film according to the orientation.^(37)^

### G. Experiments to characterize the scanner

#### G.1 Reproducibility of the response of the scanner

The reproducibility of the flatbed Epson Perfection V750 scanner response was investigated by repeatedly scanning film pieces irradiated at 0, 1, 6, 15, 35, and 70 Gy at different times: 30 min, 18 hrs, and 25 days between scans.

#### G.2 Uniformity of the response of the scanner

To determine the uniformity of the response of the scanner, films pieces were irradiated with 0, 1, 6, 15, 35, and 70 Gy and later digitized these films at 20 different positions on the bed of the scanner. The uniformity was evaluated based on the standard deviation in the response of the film placed to each position on the bed of the scanner with respect to standard deviation of central position of the film placed in central position on the bed of the scanner.

## III. RESULTS

### A. Dynamic range

**Figure 1:**
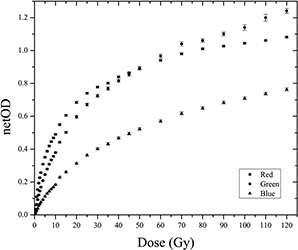
Dose response curves of EBT3 radiochromic film for the three color channels.

Figure 1 shows the dose response curves of the EBT3 radiochromic film for all three color channels from the RGB scanned images and doses ranging from 0 to 120 Gy. These curves represent the film response (net optical density, netOD) as a function of the dose delivered to the film. As indicated in the figure, the response curves of the radiochromic film scanned in the red and green channels are above the response curve of the films scanned in the blue channel; these results are consistent with those obtained for the EBT radiochromic film.^(10)^ Therefore, the signal weakly depends on the dose and strongly depends on the thickness of the active layer in the blue channel. The film response curves for the red and green channel show an intersection at 50 Gy; this value is consistent with data available in the literature.^(10)^ Also, from Fig. 1 it can be observed that there are relatively low changes of netOD for dose values greater than 30 Gy and the film response saturation starts approximately at 60 Gy. Starting at this point, the response curve for films scanned with the red channel shows a more rapid saturation than the response curve for the green channel. The response behavior of the radiochromic film to radiation could be attributed to the absorption spectrum of the active layer, which exhibits maximum absorption at approximately 635 nm (i.e., the red spectrum of visible light). Furthermore, the absorption spectrum has a lower absorption peak centered at approximately 583 nm that falls within the green visible spectrum. Because the absorption peaks found in the blue part of the visible spectrum are very small, the response of the film in the blue channel is below the response of the red and green channels.^41^, ^42^ In addition, the net optical density is a measure of the convolution of the active layer absorption spectrum, the linear CCD array sensitivity spectrum, and the emission spectrum of the fluorescent light source of the scanner.^(10)^

**Figure 2:**
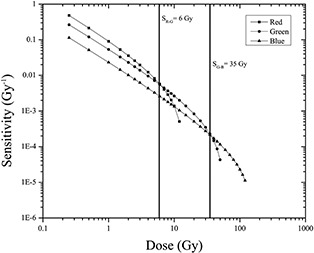
Sensitivity curves of EBT3 radiochromic film for the three color channels.

Because the response curves of the EBT3 radiochromic film do not accurately define the dynamic ranges for each color channel, the response sensitivity of the film is analyzed. The response sensitivity of the film is defined as the slope of the response curve for each dose value and is mathematically expressed as the derivative of the response curve for each dose value.^(38)^ Figure 2 shows the sensitivity curves of each color channel as a function of the delivered dose, which were used to define the dose regions of maximum sensitivity for a particular color channel. In general, the sensitivity of the response of the film decreased with the dose. Furthermore, the sensitivity depends on the color channel with which the films are scanned. Figure 2 shows two points of interest, one corresponding to the intersection of the sensitivity curves of the red and green channels ($S_{\text{RG}}=6\text{Gy}$) and the other being the intersection of the sensitivity curves of the green and blue channels ($S_{\text{GB}}=35\text{Gy}$). These points define the intervals of maximum sensitivity for each color channel. In other words, for the dose range of 0−6Gy, the film response is more sensitive when scanning with the red channel, whereas from 6−35Gy, the response is more sensitive if we scan the film with the green channel. For doses greater than 35 Gy, the sensitivity in the response of the film is maximized if the film is scanned with the blue channel. Notably, the dynamic ranges and response curves depend on the dosimetry system used, which consists of a particular model of radiochromic film, a flatbed scanner, and a dosimetry protocol.

### B. Response curves and fitting procedure

**Figure 3:**
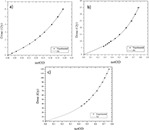
Curve fitting of EBT3 radiochromic film (a) from the red channel scanned within dose range from 0 to 6 Gy. (b) Curve fitting of EBT3 radiochromic film from the green channel scanned within dose range from 6 to 35 Gy. (c) Curve fitting of EBT3 radiochromic film from the blue channel scanned within dose range from 35 to 120 Gy.

Figure 3 shows the fitting curves for each color channel within the dose regions defined above (Results section A.). The fit parameters a, b, and c in the analytical expression given by Eq. (4) were determined for each color channel for data corresponding to the highest sensitivity range. These ranges were: from 0 to 6 Gy for the red channel, from 6 to 35 for the green channel, and from 35 to 120 Gy for the blue channel. The fitted curves coincided with the experimental values in the dose ranges for the color channel that was most sensitive.

**Figure 4:**
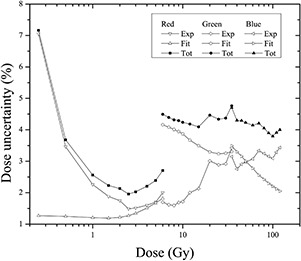
Dose uncertainty analysis for the three color channels within dose regions defined by sensitivity curves.

Figure 4 shows the analysis result of uncertainty for each color channel considering the ranges over which the response of the film was most sensitive. For radiochromic EBT3 films scanned using the red channel at doses ranging from 0 to 6 Gy, an average total uncertainty of 2.6% was obtained, representing the lower limit of lower uncertainty when using the system consisting of the EBT3 radiochromic film and the Epson Perfection V750 scanner for dosimetry. Moreover, the average total uncertainty for films scanned with the green channel in the dose range from 6 to 35 Gy was 4.3%, which is the maximum average total uncertainty. Finally, the total uncertainty in determining the dose for the blue channel was 4.1% for doses ranging from 35 to 120 Gy.

In addition, experimental uncertainty contributed the most to the calculation of the total uncertainty in the red channel, whereas greater uncertainty was associated with the fitting process in the green channel. For the blue channel, the associated uncertainty decreased with the dose. Therefore, the total uncertainty for the three color channels was less than 5%, but only the red channel exhibited a lower total uncertainty of 3%.

**Figure 5:**
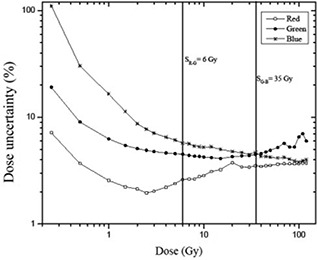
Total dose uncertainty for the three color channels defined by the sensitometric curves.

**Figure 6:**
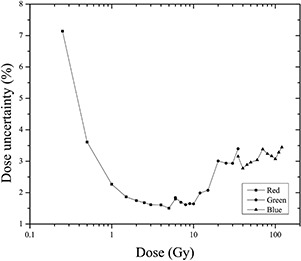
Dose resolution of the dosimetry system for the three color channels in their respective dynamic ranges.

Figure 5 shows the total dose uncertainty for the three channels from 0 to 120 Gy. It can be observed, that the three channels show the same trend. For low doses, the dose uncertainty decrease as a function of dose, nerveless for higher doses, the dose uncertainty increases not monotonically in the three channels. Figure 6 shows the same uncertainty data that is shown in Fig. 5 but only the total dose uncertainty is plotted, corresponding to the dose interval recommend for each color channel by the film manufacturer: 0‐10 Gy for red, 10‐40 Gy for green, and >40Gy for blue channel. It can be observed that the total dose uncertainty increases for the green and blue channels as a function of dose.

### C. Dose resolution of the system

Figure 6 shows the results of the uncertainty analyses for the dose resolution of the dosimetric system consisting of the EBT3 radiochromic film and Epson Perfection V750 flatbed scanner for the doses ranging from 0 to 120 Gy. The uncertainty associated with the resolution of the dosimetry system depends on the dose and the color channel used. For the red channel, the uncertainty decreases with dose, presenting with an average value of 1.8%. In contrast, for the green and blue channels, uncertainty tended to increase with the dose. However, the average uncertainty in dose for the green channel was the same as that of the red channel, 2.3%, whereas that for the blue channel was 3.1%. The results obtained in this work for the red channel are consistent with those shown in the literature for the EBT film Epson Expression 1680 Pro flatbed scanner for the red and green channels.^(5)^

### D. Film uniformity and reproducibility

**Figure 7:**
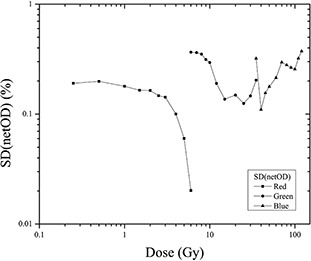
Standard deviation of the film response (net optical density) for the three color channels and the dose ranges determined in this work.

Figure 7 presents the analysis of the reproducibility of the response of the EBT3 radiochromic film irradiated from 0 to 120 Gy. This analysis was conducted for the three colors, from 0 to 6 Gy for the red channel, from 6 to 35 Gy for the green channel, and from 35 to 120 Gy for the blue channel. The figure shows the behavior of the standard deviation (reproducibility in film response) as a function of the delivered dose. For films irradiated from 0 to 6 Gy and scanned in the red channel, the figure shows that the standard deviation in the response of the EBT3 radiochromic film (net optical density) decreases with dose (i.e., the reproducibility of the film increases with dose). Conversely, for films irradiated at doses ranging from 6 to 35 Gy and from 35 to 120 Gy scanned in the green and blue channels, respectively, the reproducibility of the film did not exhibit a defined behavior. Finally, the reproducibility of the EBT3 radiochromic film was increased when the film is scanned within the red channel, with an average standard deviation in the response of the film that was lower than 0.2%. For the films scanned with the green and blue channels, the average standard deviation of the response of the film was lower than 0.3%. The literature reports a higher uncertainty in the reproducibility of the response for the radiochromic EBT2 film model, the uncertainty in the reproducibility of the response was 1.5% and less 0.6% for the EBT film model.^3^, ^5^

However, the nonuniformity of the radiochromic EBT3 film was less than 0.2% for the red channel. This result is consistent with that reported for the EBT3 film by Casanova‐Borca et al.;^(1)^ they found a nonuniformity of less than 1%. The nonuniformity of the EBT film was also less than 1%.^(5)^ However, the EBT2 film nonuniformity was 0.7%.^(3)^ For the green and blue channels, the nonuniformity had values of less than 0.3%.

### E. Relative orientation of the film

**Figure 8:**
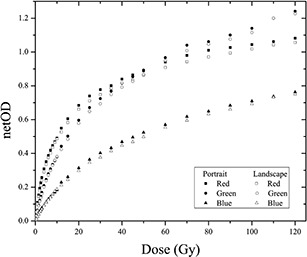
Dose response curves of EBT3 radiochromic film for the three color channels scanned in portrait and landscape mode.

Figure 8 shows the response curves of the radiochromic EBT3 films scanned in portrait and landscape mode for doses ranging from 0 to 120 Gy. Response curves were obtained for the three color channels in their respective ranges of highest sensitivity. Differences are observed in the response of the film when it was scanned in landscape mode and portrait mode in the three color channels. However, the difference in the response was greater for the red channel than for the green and blue channels. The difference in the response of the film to the red channel was 6.2% in portrait mode, whereas these differences were 2.7% and 3.3% for the green and blue channels, respectively. These differences in the response of the film have been studied and quantified in models for EBT^6^, ^43^ and EBT2^(3)^ radiochromic films. These studies concluded that the influence of the relative film orientation in the scanner is significant and, therefore, should be considered when conducting dosimetry analyses with radiochromic film. Notably, the differences in response due to the relative orientation of the scanner depend on the type of film and scanner model used. The reported differences in the response of the EBT radiochromic film are 6.2%^(5)^ and 11.5% for the EBT2 film model^(3)^ relative to the portrait orientation. However, the reported differences for the EBT3 film model was less than 4.5% with reference films scanned in portrait mode.^(1)^ Another report compares portrait and landscape film scans with an Epson Expression 1680 Pro. This work concluded that, due to the influence of rotation on optical densities, the results showed a minimum reduction in OD of 3.9% for EBT2 and EBT3. Also, the study shows a lower dose intensified this effect with a peak relative difference of 7.1% for the 50c Gy dose level.^(44)^

**Figure 9:**
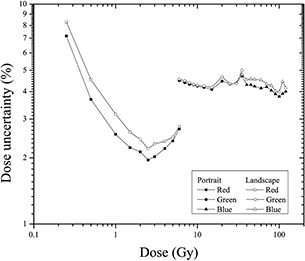
Dose uncertainty analysis for the three color channels scanned in portrait and landscape mode.

Figure 9 shows the uncertainty analyses for the relative orientation of the film during scanning. This analysis was performed for the radiochromic EBT3 films by scanning them in portrait and landscape mode using the three color channels for dose intervals that maximized the sensitivity of the response. Overall, the results show that the uncertainty in determining the dose is less when the films are scanned in portrait mode than when they are scanned in landscape mode. This difference was observed for all three color channels. The average uncertainty in dose for the red channel was 3%, whereas that for the green and blue channels was 4.4%. Moreover, the average uncertainty values for films scanned in portrait mode were 2.6%, 4.3%, and 4.1% for the red, green, and blue channels, respectively.

### F. Reproducibility of the response of the scanner

The reproducibility of the Epson Perfection V750 flatbed scanner was studied for time intervals of 30 min, 18 hrs, and 25 days between scans. For all color channels and times, the uncertainty in the dose was less than 0.3%, indicating that the reproducibility of the scanner readings was good. An uncertainty of less than 1% was reported EBT films irradiated at 2 Gy.^(5)^

### G. Uniformity of the response of scanner

**Figure 10:**
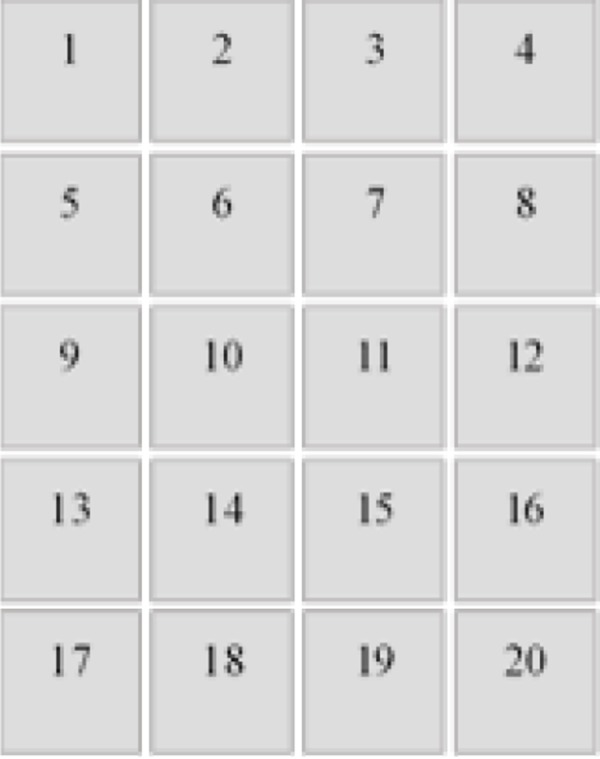
EBT3 radiochromic film positions on the film scanner bed.

EBT3 radiochromic films were digitized at 20 different positions on the bed of a scanner, as shown in Fig. 10. The results of our uncertainty analysis show that the uncertainty obtained in determining the dose is higher when placing the film at a position other than the center of the scanner bed than when placing the film at the center. This difference was observed for all tested doses. The differences in the response of the films placed farthest from the center of the scanner bed (positions 1, 4, 17, and 20) were 5% for the red channel, 7% for the green channel, and 10% for the blue channel with respect to the center position. Conversely, the differences in the response of the films placed near the center of the scanner (positions 6, 7, 10, 11, 14, and 15) were 2%, 3%, and 4.5% for the red, green, and blue channels, respectively. As shown in the figure, the increase in the uncertainties of the central position of the scanner bed with respect to any other position is small. For the Epson Expression 1680 Pro flatbed scanner evaluated with radiochromic EBT film, the difference in the response was 8% using the red channel. However, for the Epson Perfection V700 flatbed scanner and radiochromic EBT2 film, the contribution of the poor positioning of the film on the scanner bed to the total uncertainty was found to be 1.6%.

## IV. DISCUSSION

This study examined several intrinsic characteristics of the dosimetry system consisting of radiochromic EBT3 film and the Epson Perfection V750 flatbed scanner that contribute to the overall uncertainty in dose determination. Furthermore, the uncertainty analysis was performed to quantify this contribution. To this end, we investigated the behavior of the radiochromic film (net optical density) as a function of the delivered dose, as well as the standard deviation associated with the response. The response of the radiochromic EBT3 film (net optical density) corroborated the results reported by Devic et al.^(10)^ for radiochromic EBT film and doses ranging from 0 to 100 Gy. In addition, the behavior for doses ranging from 0 to 40 Gy corroborated the results reported by Casanova‐Borca et al.^(1)^ for EBT3 film.

It is important to remember that the uncertainty in determining the dose depends not only on our dosimetry system but also on the measurement protocol used. In our case, the behavior of the radiochromic film (net optical density) as a function of the delivered dose as well as the standard deviation associated with the response. Under these conditions, the dynamic ranges for the red, green, and blue channels are 0−6Gy,6−35Gy, and 35−120Gy, respectively. Moreover, Casanova‐Borca found that the red channel exhibits a greater response for doses up to 10 Gy and the green channel exceeds the response of the red channel for doses above 10 Gy. Therefore, the green channel should be used for doses up to 40 Gy. These results were obtained with an Epson Expression 10000XL flatbed scanner using a third‐degree polynomial fit. Furthermore, at study by Devic and coworkers of a dosimetry system consisting of radiochromic EBT film and an Epson Expression 1680 flatbed scanner at doses ranging from 0 to 100 Gy yielded optimized ranges of 0−4Gy for the red channel, 4−50Gy for the green channel, and above 50 Gy for the blue channel.

It is noteworthy that an important parameter in uncertainty analysis is the flatbed scanner temperature. The analysis within this study does not try to evaluate uncertainty due to the flatbed scanner temperature, but is necessary an estimation of this source of uncertainty. The flatbed temperature can become an important variable when making many successive scans. Buchauer et al.^(45)^ shows the readout difference in film is strongly dependent on readout light spectral characteristic, irradiation dose, and temperature. In addition, that the characteristic temperature behavior patterns are present for each color channel of a flat bed scanner.

Table 1 summarizes the characteristics of the dosimetry system, radiochromic EBT3 film, and flatbed scanner analyzed in this paper. In addition, shows the contribution of each of these features to the total uncertainty. These uncertainties were calculated for each color channel: red, green, and blue. In general, the values of the uncertainties were lower for the red channel than for the green and blue channels. However, for all three color channels, the largest contribution to the total uncertainty was due to the fitting procedure, the dose resolution of the system, the relative orientation of the film, and the homogeneity on the bed of the scanner. Therefore, when the radiochromic film is scanned, care should be taken to place it at the center of the scanner bed because the light from the lamp is not emitted evenly,^(31)^ and this orientation with respect to the scanner should be noted. Moreover, if we rotated the recommended film 90° to the scan direction (portrait), the uncertainty of the dose significantly increased because the polymer chains are producing a network effect to interfere with the electromagnetic radiation from of the light source of the scanner.^(46)^ One of the characteristics that make radiochromic films ideal candidates for the dosimetry of unconventional fields is their high spatial resolution (< 1200 lines/mm).^(47)^ However, the resolution in the dose is limited by the optical reading system, in this case, the spatial resolution of the flatbed scanner. As the results show, the resolution of the dosimetry system significantly contributes to the overall uncertainty in dose. Conversely, the uniformity and reproducibility of the radiochromic film and reproducibility of the response of the scanner contribute less than 1% to the overall uncertainty in dose. Nevertheless, a strict protocol for the handling and use of radiochromic films must be followed to minimize this uncertainty. From Fig. 6, if it is considered the allowed total uncertainty in delivered dose for radiation therapy of 5%,^(48)^ the minimum dose that can be measured with an overall uncertainty less than 5% is 0.4 Gy. On the other hand, if it is considered that suggested total uncertainty in the delivered dose for radiosurgery of 2%,^(30)^ the minimum dose that can be measured with an overall uncertainty less than 5% is 0.8 Gy.

Table 1 shows the total uncertainty in the measured dose based on the intrinsic characteristics evaluated in this work without considering the relative orientation of the film and scanner homogeneity because international recommendations suggest a rigorous control when the films are scanned as to the orientation and position in the scanner. Specifically, the uncertainty for the red channel was 3.2%. Accordingly, our results are consistent with those reported in the literature. However, the overall uncertainties in the dose for the green and blue channels were 4.9% and 5.2%.

**Table 1:** Summary of dose uncertainties in percentage (%).

*Characteristic*	*Red Channel*	*Green Channel*	*Blue Channel*
Response curves and fitting procedure	2.6	4.3	4.1
Dose resolution of the system	1.8	2.3	3.1
Film reproducibility	0.2	0.3	0.3
Film uniformity	0.2	0.3	0.3
Relative orientation of the film	6.2	2.7	3.3
Reproducibility of the response of the scanner	0.3	0.3	0.3
Homogeneity on the bed of scanner	2.0	3.0	4.5
Total Uncertainty^a^	3.2	4.9	5.2

^a^ Without considering relative orientation of the film and homogeneity on the bed of the scanner.

## IV. CONCLUSIONS

In this study, the dosimetry system consisting of EBT3 radiochromic film and an Epson Perfection V750 scanner for doses ranging from 0 to 120 Gy using three color channels was evaluated and analyzed, and an uncertainty analysis of the dose was performed to study certain intrinsic characteristics of film dosimetry. According with our uncertainty analysis, it is notable that the higher uncertainties found were: 1) the relative orientation of the film, 2) the uniformity of response of the scanner, and 3) the fitting procedure in decreasing importance. However, when taking into account international recommendations on handling of the films when they are analyzed in a scanner, which state that one must have strict control of the position and orientation of the film, the total uncertainties decrease considerably. Therefore, the radiochromic films can be used in a wide branch of applications in a 0−120Gy useful range considering their associated uncertainties as a function of dose.

## COPYRIGHT

This work is licensed under a Creative Commons Attribution 3.0 Unported License.

## Supporting information

Supplementary Material

Supplementary Material

Supplementary Material
