# Supplementary material for: Evaluation of the uncertainty in an EBT3 film dosimetry system utilizing net optical density
Source: J Appl Clin Med Phys. 2016 Sep 8;17(5):466–81. doi: 10.1120/jacmp.v17i5.6262 (PMC5874103; doi:10.1120/jacmp.v17i5.6262)
Supplement: Supplementary file 1 — Supplementary Material [file ACM2-17-466-s001.docx]

Evaluation of the intrinsic characteristics of a radiochromic EBT3 film dosimetry system and its contribution to uncertainty in dose

**E.Y. León Marroquin^1^, J.A. Herrera González^2^, M.A. Camacho López^1^, J.E. Villarreal Barajas^3^, O.A. García-Garduño^2^**

^1^Laboratorio de Fotomedicina, Biofotónica y Espectroscopia Láser de Pulsos Ultracortos, Facultad de Medicina, Universidad Autónoma del Estado de México, Jesús Carranza y Paseo Tollocan s/n. Toluca, México, C.P. 50120

^2^Laboratorio de Física Médica & Unidad de Radiocirugía, Instituto Nacional de Neurología y Neurocirugía, Mexico City, 14269 México

^3^Departamento de Oncología & Departamento de Física y Astronomía, Universidad de Calgary, Calgary, Alberta, Canada

^3^Laboratorio de Física Médica, Instituto Nacional de Neurología y Neurocirugía, Mexico City, 14269 México

**Corresponding author:**

Olivia Amanda García Garduño, Ph.D.

**Address and contact information:**

*Laboratorio de Física Médica*

*Instituto Nacional de Neurología y Neurocirugía*

*Insurgentes Sur 3877, La Fama*

*Tlalpan, 14269*

*Mexico City*

*México*

Tel: +52 5556063822 × 4471, 5021

e-mail: oagarciag@innn.edu.mx, amanda.garcia.g@gmail.com

Web page: [http://www.innn.salud.gob.mx](http://www.innn.salud.gob.mx/)
