# Supplementary figures and images for: Evaluation of the uncertainty in an EBT3 film dosimetry system utilizing net optical density
Source: J Appl Clin Med Phys. 2016 Sep 8;17(5):466–81. doi: 10.1120/jacmp.v17i5.6262 (PMC5874103; doi:10.1120/jacmp.v17i5.6262)

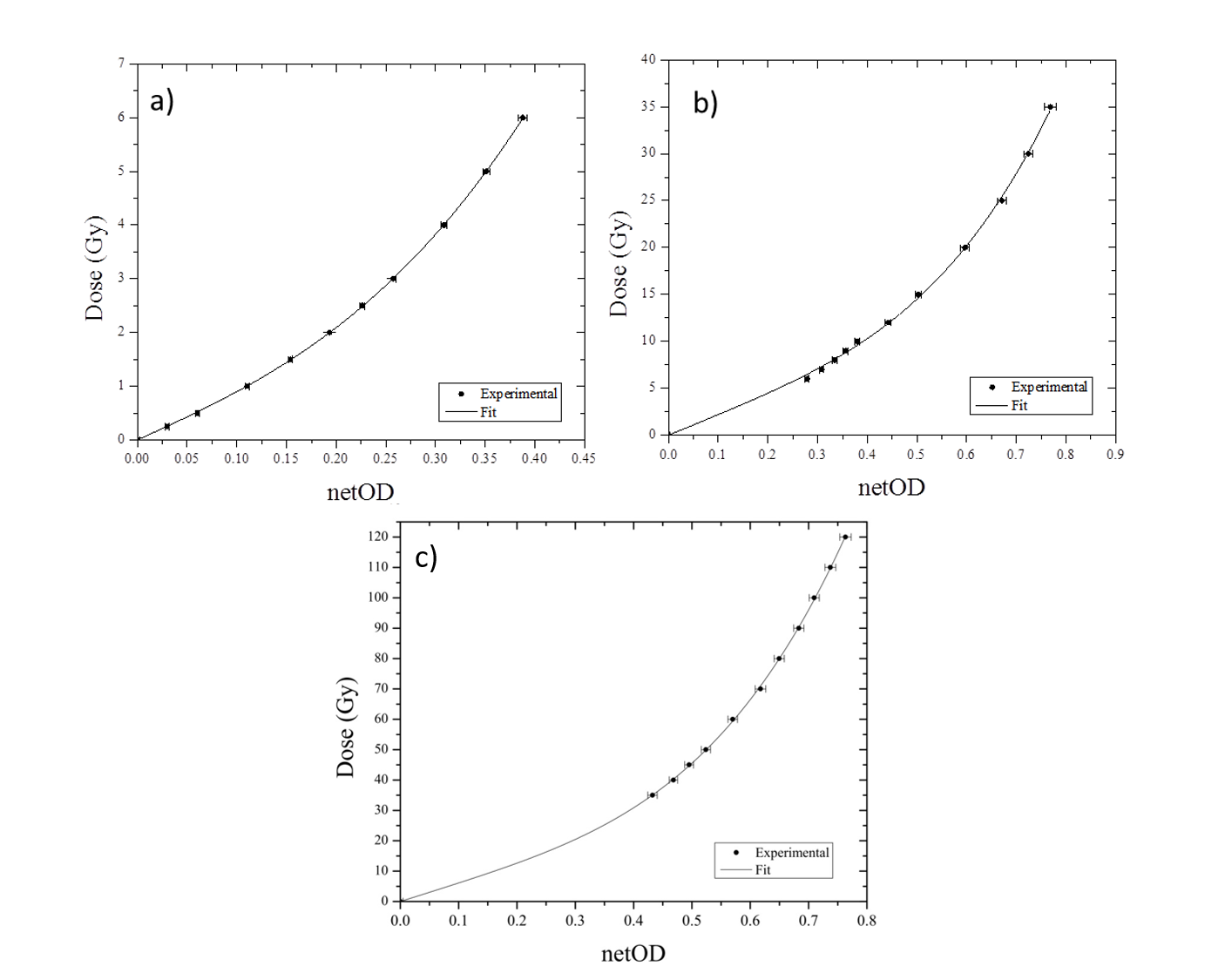

Supplement: Supplementary file 3 — Supplementary Material [file ACM2-17-466-s003.png]
